# Predictivity of Biochemical Markers on Aetiology and Length of Hospitalisation in Acute Pancreatitis

**DOI:** 10.7759/cureus.11989

**Published:** 2020-12-09

**Authors:** Umasankar Mathuram Thiyagarajan, Amirthavarshini Ponnuswamy, Rhys Thomas

**Affiliations:** 1 Hepatobiliary and Pancreatic Surgery, Addenbrooke's Hospital, Cambridge University Hospitals NHS Foundation Trust, Cambridge, GBR; 2 Family Medicine, Kent, Surrey and Sussex Deanery, Epsom, GBR; 3 General Surgery, Croydon University Hospital, Thornton Heath, GBR

**Keywords:** acute pancreatitis, alanine aminotransferase, alkaline phosphatase, gallstones, bilirubin, lipase

## Abstract

Background

Acute pancreatitis (AP) is a common cause of emergency hospital admission. Predictive value of biochemical markers including alanine aminotransferase (ALT), alkaline phosphatase (ALP), bilirubin and lipase on pancreatitis has not been fully established. This study aimed to assess the role of ALT, ALP, bilirubin and lipase levels at admission on predicting the aetiology and length of hospital stay in AP. This study also assesses quantitative high lipase as a predictor of gallstone pancreatitis (GP).

Methods

All patients above the age of 18 with a diagnosis of AP between October 2016 - 2017 were included in our study. The exclusion criteria were patients with a known history of pancreatitis or biliary disease/bile duct stones and pregnancy. This is a retrospective study performed from a prospectively collected electronic patient database at our hospital.

Results

Among the 143 patients with AP, 50 patients were diagnosed with gallstone pancreatitis (GP) and the remaining of 93 patients suffered non-gallstone pancreatitis (NGP). Mean ALT level was significantly higher in gallstone pancreatitis (237 ± 351 IU) compared to non-gallstone pancreatitis (107 ± 162 IU; P = 0.005). ALP level was numerically high in GP (151.5 ± 186) compared to NGP (138 ± 105 IU; P = 0.64). Similar results in bilirubin level also noted in GP (35.5 ± 24.5) comparing to NGP (20.7 ± 79.6 µmol/L; P = 0.09).

Raised ALT (9.3 ± 8.2 versus 3 ± 2.19 days), bilirubin (8.5 ± 2.5 versus 6.9 ± 1.19 days) and ALP levels (6.26 ± 6.1 versus 3.5 ± 10 days respectively; P = 0.05) were associated with longer hospitalisation in GP comparing to NGP. The lipase level more than 10 times the upper reference level (10-URL) was found to be associated with GP (39/50) than NGP (54/93; P = 0.027).

Conclusion

Raised ALT, high lipase of 10 URL levels were associated with gallstone pancreatitis. In gallstone pancreatitis, patients with high ALT, bilirubin and ALP levels had longer hospital stay.

## Introduction

Acute pancreatitis (AP) is a known common cause of acute abdomen, usually needing an emergency hospital admission. Worldwide, the incidence of AP varies between 4.9 and 73.4 cases per 100,000 [1-2]. In UK, the approximate incidence ranges from 15 to 42 cases per 100,000 population [3, 4]. In 2009, AP was the most common gastroenterology discharge diagnosis with a cost of 2.6 billion dollars in the Unites States [1]. Though there was a slight reduction in the mortality at the end of last century, the incidence is on steady increase [3].

Biochemical markers in pancreatitis

Although AP is a known reason for hospital admission, reaching the diagnosis can be difficult due to the lack of an effective and simple blood test with high sensitivity. The commonly used markers are serum amylase, lipase, trysinogen-2, and activation peptide of carboxypeptidase B [5-7].

Alanine aminotransferase (ALT) is present primarily in liver cells. In liver disease, serum ALT was shown to be elevated even before the clinical signs and symptoms of the disease appear [8]. Predictivity of transaminases, alkaline phosphatase (ALP) and bilirubin in gallstone pancreatitis (GP) is still debatable. There were conflicting findings that have been reported in the past and high quality evidence is lacking [7, 9-12].

There are evidences to support that lipase is more specific to pancreas and also cost-effective [13-14]. But non-specific hyperamylasemia and hyperlipasemia have been reported in non-pancreatitis causes like diabetic ketoacidosis. The incidence of nonspecific hyperamylasemia is up to one-fourth of cases in diabetic ketoacidosis [15]. It is also known that serum amylase and lipase can also be elevated in other non-pancreas causes including mesenteric infarction, perforated viscus, trauma, acute appendicitis, inflammatory bowel disease, bowel obstruction, fat embolism, liver, renal failure and hypertriglyceridemia [16].

The UK guidelines for the management of acute pancreatitis recommend serum lipase for diagnosing acute pancreatitis due to the fact that it has longer half life and only released from the pancreatic acinar cells [17]. In contrary, American Gastroenterology Association recommends both amylase and lipase levels for diagnosing acute pancreatitis [18].

## Materials and methods

Patients with the diagnosis of AP between October 2016-2017 were included in this study. Due to the nature of the study, neither ethical committee approval nor patients’ consent was required in line with our hospital policy. Among the 143 patients with acute pancreatitis, 50 patients diagnosed with GP and remaining of 93 patients suffered non-gallstone pancreatitis (NGP). All relevant parameters including demography, laboratory tests, radiological investigations and length of hospital stay (LOS) were retrieved from a prospective electronic patient database.

All our patients had serum lipase (reference range 0-50 U/l), full blood count, bone profile, liver function tests including ALT, ALP, bilirubin, urea and electrolytes and lactate dehydrogenase when attending the hospital. All patients with acute pancreatitis in our study have the aetiology established. The exclusion criteria were known patients with previous gallstone pancreatitis or bile duct stones/biliary disease and age below 18 years.

All of our patients in this study underwent abdominal ultrasonography to rule out gallstone disease; patients with deranged liver function, dilated bile duct, or high suspicion of bile duct stones had magnetic resonance cholangio-pancreatography (MRCP) in line with our local hospital guidelines. Computerised tomography (CT) scan was performed in all patients with severe AP by using modified Imrie score of three and when there was a diagnostic uncertainty [19]. Endoscopic retrograde cholangio-pancreatography (ERCP) was performed within the same hospital admission in all patients with jaundice, bile duct stones and cholangitis.

Statistical analysis

Data were recorded in an Excel spread sheet (Microsoft, Redmond, WA, USA) and GraphPad Instat version 3.06 (GraphPad Software, Inc, San Diego, CA, USA). Descriptive statistics for continuous variables were recorded as Mean ± Standard Deviation (SD) or Median (range) according to whether or not they were normally distributed. Normality testing was performed using the Kolmogorov-Smirnov test. Comparison between groups was performed with Student’s t-test for parametric variables and the Mann-Whitney test for non-parametric variables. Categorical variables were analysed using the chi-squared test with Yates correction or Fisher’s exact test. Statistical significance was defined as P < 0.05.

## Results

Among the 143 patients with acute pancreatitis, 50 of them (50/143- 35%; Table 1) have been diagnosed with gallstone pancreatitis and remaining (93/143- 65%; Table 1) were of non-gallstone pancreatitis. Interestingly majority of patients in our study population were of non-gallstone pancreatitis aetiology. Within the gallstone pancreatitis group, 21 were males and 29 were females; similarly 62 males, 31 females in non-gallstone pancreatitis group (P = 0.07). There was no difference in age between gallstone pancreatitis compared with non-gallstone pancreatitis (P = 0.90, Table 1). There was no difference noted in white blood cell count (WBC), C-reactive protein (CRP), American Society of Anesthesiologists (ASA) 3 grade, MRCP and CT scans between GP and NGP (Table 1). As expected, more patients in the GP had ERCP (20/50 versus 5/93; P = 0.0001, Table 1) for bile duct obstruction. Table 1 shows the incidence of severe acute pancreatitis between the GP and NGP (19/50 versus 44/93; P = 0.25). Patients in GP arm had longer hospitalisation when compared to NGP (6.6 ± 6 versus 3.4 ± 9.3; P = 0.001, Table 1).

**Table 1:** Demographic parameters Ѱ – Values in Mean ± Standard Deviation WBC: White Blood Cell count; CRP: C-Reactive Protein; LOS: Length of Stay at Hospital; MRCP: Magnetic Resonance Cholangio-Pancreatography; CT: Computerised Tomography; ASA: American Society of Anesthesiologists physical status classification.

	Gallstone Pancreatitis (n = 50)	Non-Gallstone Pancreatitis (n = 93)	P-Value
Age (years)^ Ѱ^	52 ± 19	51.9 ± 15.9	0.90
Gender (M:F)	21:29	62:31	0.07
ASA 3 or more	28/50	62/93	0.27
WBC at admission^ Ѱ^	11.89 ± 3.5	11.3 ± 5.01	0.24
CRP at admission^ Ѱ^	30.4 ± 73	47.6 ± 79	0.25
MRCP scan	33/50	34/93	0.85
CT scans	17/50	59/93	0.85
Severe AP	19/50	44/93	0.25
ERCP	20/50	5/93	0.0001
LOS^ Ѱ^	6.6 ± 6	3.4 ± 9.3	0.001
30 Days Mortality	0/50	2/93	0.54

Predictivity of biochemical markers on aetiology of pancreatitis

Our study showed that high ALT, bilirubin, ALP and lipase at admission were associated with gallstone pancreatitis. The sensitivity and specificity of the ALT was 76 (0.61 to 0.86; 95% CI) and 46% (0.35 to 0.56; 95% CI), respectively. The positive predictive value was 0.43 (0.32-0.56; 95% CI).

**Figure 1:**
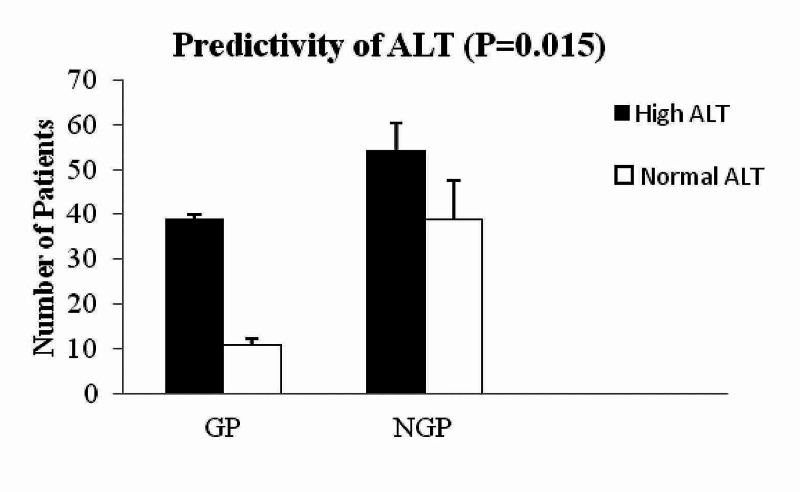
ALT level in GP versus NGP Mean ALT level was high in GP compared to NGP (P = 0.015) ALT: Alanine aminotransferase; GP: Gallstone pancreatitis; NGP: Non-gallstone pancreatitis.

**Figure 2:**
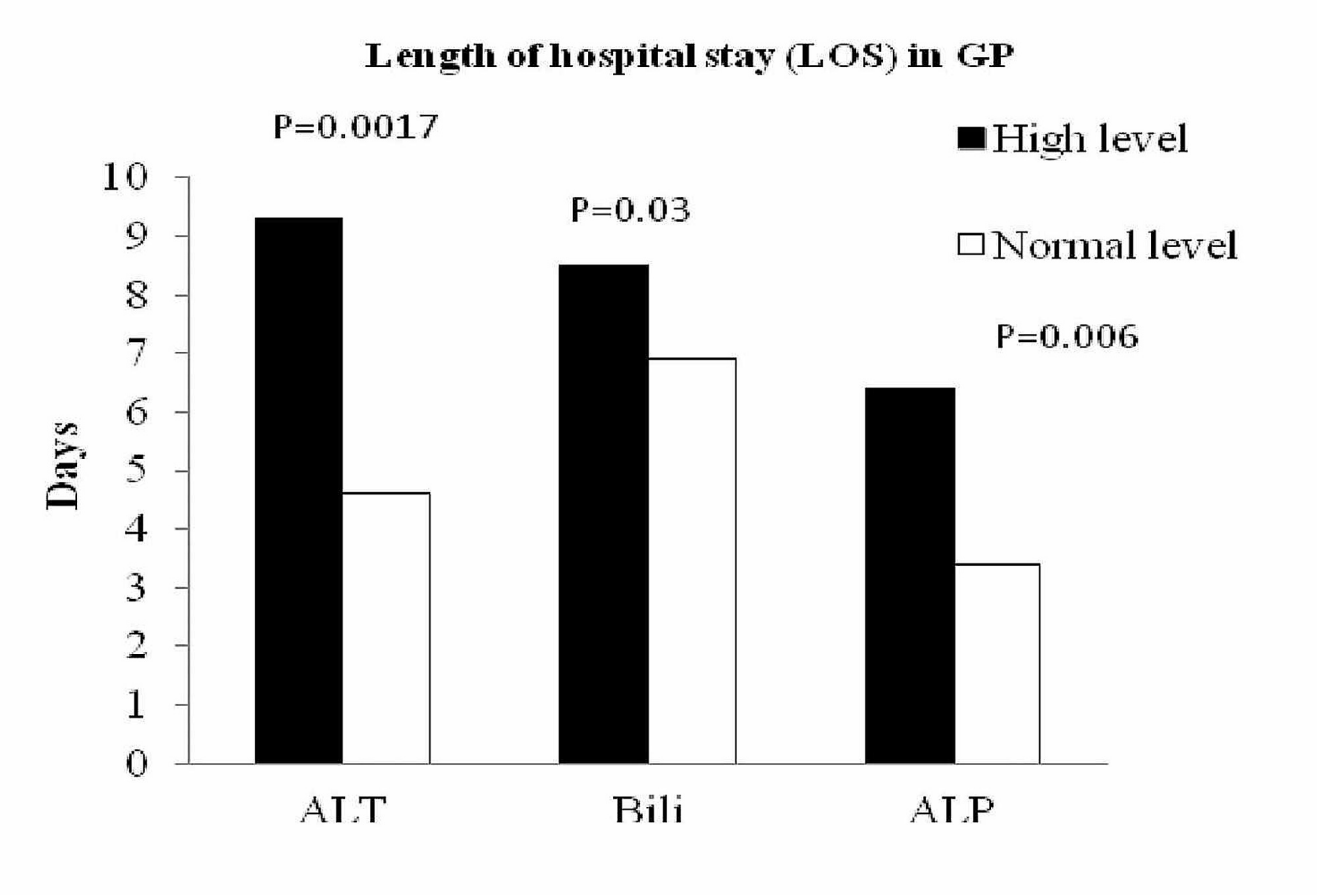
Impact of biochemical markers on hospital stay in GP Patients with elevated ALT, bilirubin and ALP had longer hospital stay in GP. ALT: Alanine aminotransferase; ALP: Alkaline phosphatase; GP: Gallstone pancreatitis.

The ALT level was significantly higher in gallstone pancreatitis (237 ± 351 IU versus 107 ± 162 IU; P = 0.005, Figures 1, 2) compared to non-gallstone pancreatitis. Though 60 of 93 patients with NGP had abnormal ALT, the mean level was found to be less when compared to GP. ALP (151.5 ± 186 versus 138 ± 105 IU; P = 0.64), bilirubin levels (35.5 ± 24.5 versus 20.7 ± 79.6 µmol/L; P = 0.09) did not show difference between gallstone pancreatitis compared with non-gallstone pancreatitis.

Lipase level more than 10 times the upper reference level (10-URL) was found to be associated with GP group (39/50 versus 54/93; P = 0.027) where the 5-URL (45/50 versus 71/93; P = 0.07) is not. Further analysis showed a 10-URL shows a sensitivity of 0.78 (0.64-0.88; 95% CI), specificity of 0.41 (0.31-0.52; 95% CI), positive predictive value of 0.41 (0.31-0.53; 95% CI).

Predictivity of biochemical markers on length of stay at hospital (LOS)

Among the GP cohort, patients with elevated ALT level had longer hospitalisation compared to the patients with normal ALT (9.3 ± 8.2 versus 3 ± 2.19 days; P = 0.0017, Figure 2). Similar findings were noted with elevated bilirubin (8.5 ± 2.5 versus 6.9 ± 1.19 days; P = 0.03) and ALP (13.5 ± 10.1 versus 6.26 ± 6.1 days; P = 0.006, Figure 2) with length of hospitalisation in GP.

**Figure 3:**
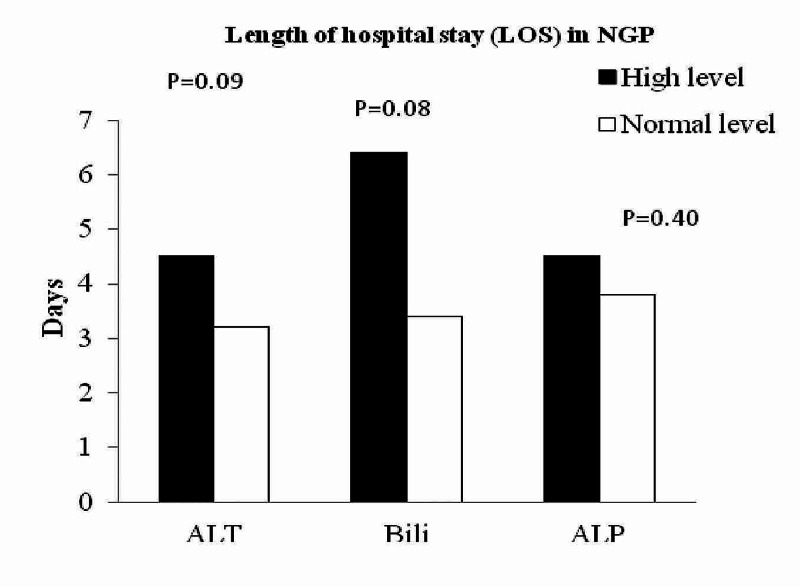
Impact of biochemical markers on hospital stay in NGP Patients with elevated ALT, bilirubin, and ALP did not have longer hospital stay in NGP (P = NS). NGP: Non-gallstone pancreatitis; ALT: Alanine aminotransferase; ALP: Alkaline phosphatase.

In NGP, this effect of elevated ALT versus normal ALT on the length of hospital stay was not observed (3.21 ± 2.28 versus 4.6 ± 4.2 days; P = 0.09, Figure 3). Patients with elevated bilirubin and ALP levels were also not found to have longer hospital stay (6.4 ± 5.7 versus 3.41 ± 2.3; P = 0.08 and 4.58 ± 2.1 versus 3.83 ± 4.1; P = 0.40 respectively, Figure 3).

**Figure 4:**
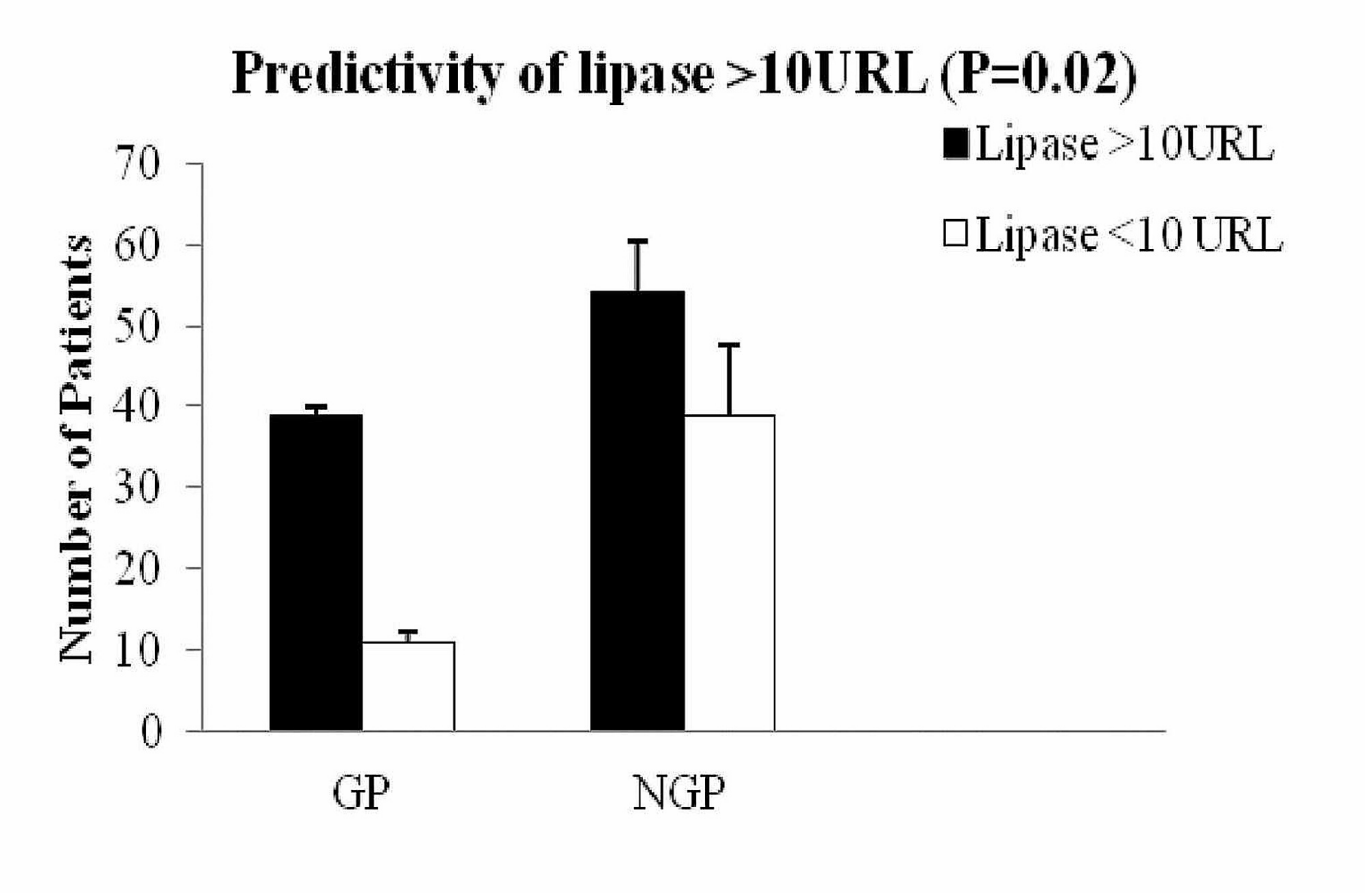
Predictivity of lipase >10 URL in GP versus NGP Patients with lipase >10 URL were more in GP comparing to NGP (P = 0.02). GP: Gallstone pancreatitis; NGP: Non-gallstone pancreatitis.

Further analysis of the data showed significantly high lipase was associated with gallstone pancreatitis compared with non-gallstone pancreatitis (2663 ± 3305 IU versus 1405 ± 2446 IU; P = 0.001, Figure 4). Mortality at 30 days between the gallstone pancreatitis versus non-gallstone pancreatitis was not different (0/50 versus 2/93; P = 0.54; Table 1).

## Discussion

Reaching the diagnosis of acute abdomen can be daunting when a surgeon needs to rule out causes that require immediate surgical intervention. There can be confusion on reaching the diagnosis due to the time lag from onset of symptoms to presentation at hospital [20]. When the diagnosis of acute pancreatitis has been made, it becomes crucial to establish the aetiology as this would guide the next steps of definitive treatment. Although the immediate management of AP is the same, gallstone pancreatitis clearly needs an early plan for an intervention.

The UK guideline recommends all patients with gallstone pancreatitis should undergo definitive management of gallstones during the same hospital admission, unless a plan is in place for definitive treatment [21]. It also recommends an urgent therapeutic endoscopic retrograde cholangiopancreatography (ERCP) to be performed in patients with suspected or proven gallstone aetiology when there is a predicted or actual severe pancreatitis, or when there is cholangitis, jaundice, or a dilated common bile duct [21]. Though American gastroenterological association guidelines do not recommend routine use of ERCP unless there is cholangitis [22]. Hence for the above reasons, establishing the aetiology is crucial in gallstone pancreatitis. In our study, more patients in GP arm had ERCP for bile duct stone; five out of 93 patients in NGP also needed ERCP for cholangitis/biliary obstruction.

Establishing the role of biochemical markers in predicting gallstone aetiology at admission would be of immense value in the management of pancreatitis. Previous studies have shown that amylase [23], serum ALP, total bilirubin, and lipase levels were significantly higher in gallstone pancreatitis [10].

Our study showed that elevated level of ALT has been associated with GP (Figure 1) and appeared to have a predictive value in GP. However, with elevated bilirubin, ALP does have any predictive value neither on GP nor on NGP. More interestingly, elevated level of ALT, bilirubin, and ALP was associated with longer hospitalisation in GP (Figure 3). However, this association was not seen in NGP patients with elevated ALT, bilirubin, and ALP. This is a very interesting finding in our study and has not been reported before in the literature. Moreover, lipase of 10-URL showed a high sensitivity of 0.78 (0.64-0.88; 95% CI) in diagnosing GP. To date, there is no similar report of 10 URL lipase and its predictivity of GP, hence this needs further investigation to confirm our findings.

These above results were in line with the previous meta-analysis that showed ALT level predicated gallstone pancreatitis [24]. Further analysis showed proportionately more males in non-gallstone pancreatitis group (62/93, Table 1) although it did not reach statistical significance (GP-21:23 versus NGP-62:31 P = 0.07). However, surprisingly no gender difference was noted in the gallstone pancreatitis group in contrary to a historical belief of female preponderance.

Although Güngör et al. showed a high ALP, bilirubin, amylase, and lipase may be used on diagnosing GP, and unfortunately this study had its inherent weakness too [10]. This study was performed with a retrospective data spanning over 15 years and hence the possibility of potential biases.

There are few limitations in our study. First, the sample size in our study was small and of retrospective nature but prospectively captured information from our electronic data has been utilised here. Secondly, though we performed the blood investigations at admission, 24 hours till discharge, we included levels of inflammatory markers for analysis up to 72 hours due to the database limitations. Thirdly, we have excluded the use of antibiotics in our study. Despite these limitations, there was strength in our study too. We have also retrieved the numbers of patients with severe pancreatitis, numbers of ERCPs, reviewed imaging, length of stay in hospital and mortality. We feel that our findings are significant but need further studies to replicate our results.

## Conclusions

Rapid diagnosis of acute pancreatitis has become possible in recent times but challenge still exists in identifying the aetiology and predicting longer hospitalisation. From our study in acute pancreatitis, elevated level of ALT, bilirubin and ALP was associated with longer hospitalisation in gallstone pancreatitis. Moreover, lipase level of 10-fold upper reference level appears to be predicting gallstone pancreatitis. These findings could have substantial potential on reducing morbidity and mortality in patients with gallstone pancreatitis. Our study reiterates the need for further studies with large sample size to establish the role of biochemical markers in predicting gallstone pancreatitis.
